# Mo-La_2_O_3_ Multilayer Metallization Systems for High Temperature Surface Acoustic Wave Sensor Devices

**DOI:** 10.3390/ma12172651

**Published:** 2019-08-21

**Authors:** Siegfried B. Menzel, Marietta Seifert, Abhinav Priyadarshi, Gayatri K. Rane, Eunmi Park, Steffen Oswald, Thomas Gemming

**Affiliations:** 1 Leibniz IFW Dresden, Helmholtzstraße 20, 01069 Dresden, Germany; 2 TU Dresden, Institute for Materials Science, 01069 Dresden, Germany

**Keywords:** SAW sensors, interdigital transducer material, high-temperature stability, dispersion strengthening, Mo-La_2_O_3_ multilayers

## Abstract

Developing advanced thin film materials is the key challenge in high-temperature applications of surface acoustic wave sensor devices. One hundred nanometer thick (Mo-La${}_{2}$O${}_{3}$) multilayer systems were fabricated at room temperature on thermally oxidized (100) Si substrates (SiO${}_{2}$/Si) to study the effect of lanthanum oxide on the electrical resistivity of molybdenum thin films and their high-temperature stability. The multilayer systems were deposited by the magnetron sputter deposition of extremely thin (≤1 nm) La interlayers in between adjacent Mo layers. After deposition of each La layer the process was interrupted for 25 to 60 min to oxidize the La using the residual oxygen in the high vacuum of the deposition chamber. The samples were annealed at 800 ${}^{\circ }$C in high vacuum for up to 120 h. In case of a 1 nm thick La interlayer in-between the Mo a continuous layer of La${}_{2}$O${}_{3}$ is formed. For thinner La layers an interlayer between adjacent Mo layers is observed consisting of a (La${}_{2}$O${}_{3}$-Mo) mixed structure of molybdenum and nm-sized lanthanum oxide particles. Measurements show that the (Mo-La${}_{2}$O${}_{3}$) multilayer systems on SiO${}_{2}$/Si substrates are stable at least up to 800 ${}^{\circ }$C for 120 h in high vacuum conditions.

## 1. Introduction

The development of advanced metallization–substrate material systems with improved properties in the high-temperature range and their comprehensive characterization have become predominant topics in the development of wireless surface acoustic wave (SAW) temperature sensors in recent years. Nevertheless, aside from material developments used at the laboratory level with a thermal stability up to 900 ${}^{\circ }$C for a relatively short time period there is no material system in the market yet with sufficient lifetime and reliability for temperatures above 600 ${}^{\circ }C$ in harsh environmental conditions which meets all the requirements for the SAW sensor chips and the sensor antennas. Besides this, the piezoelectric substrate material, the metallization for the interdigital transducers (IDT) and additional functional layers like diffusion barriers (to the substrate) or cover layers (to protect the sensor surface against ambient or packaging elements), as well as the sensor antenna have to maintain their functionality during application at those temperatures for a long time and with high reliability. In the high temperature range, the most critical criteria for the applicability of the IDT material is its low electrical resistance and high thermal stability especially with respect to mechanical properties and creep. In order to be able to simulate and design dedicated SAW structures all SAW relevant material parameters and their temperature dependence need to be known. This means, degradation processes due to a stress-induced material transport (acoustomigration), a relaxation of mechanical stress, an irreversible change in the electric resistance or a thermally-induced change in the constitution of the materials like phase transitions, cracking or blistering and so forth are not acceptable in the structured film-substrate material system. In order to use a SAW device as a sensor a strong correlation between the measured frequency characteristics and the observed parameter is essential for a highly reliable operation of the SAW device. Depending on the acoustic power density, the operation frequency and the temperature including thermal cycling conditions several aspects for the material development have to be considered to get a strong correlation between the measured frequency characteristics (the measured variable) and the temperature of the device (the target variable). Thermally activated effects like drift-diffusion, agglomeration, creep and dislocation movement in the finger material under the conditions of high temperature and extremely high cyclic mechanical load (MHz to GHz frequency range) are the most relevant damaging effects at operation temperatures above 30 to 50% of T${}_{\mathrm{melt}}$ (T${}_{\mathrm{melt}}$: melting point of the metallization). In addition, chemical effects such as corrosion and oxidation also have to be considered since they have an influence on the frequency behavior and applicability of the SAW sensor devices. With regard to their thermal stability, materials and material systems with a high melting point like refractory metals, alloys and temperature stable intermetallic phases without any phase transition within the operation temperature range are generally favored for such applications.

Typical metallization materials for the fabrication of IDTs like Al, Cu, Ag or Au or alloys or multilayers of them are mostly damaged in long-term and/or cyclic operation of SAW devices under harsh environmental conditions [1,2,3,4,5]. Therefore, other IDT material concepts have to be evaluated that not only deliver high thermo-mechanical stability but also good electrical properties besides chemical resistance to corrosion and oxidation. Metallizations based on noble materials like Pt, Ir, Pd, Re and Rh are alternatives and often reported in the literature [6,7,8,9,10,11,12]. Unfortunately, pure thin films of these materials with a thickness lower than 150 nm are typically damaged by agglomeration due to their weak chemical affinity to the substrate and dewetting effects. Nevertheless, alloying, dispersion hardening and adapted adhesion and covering layers can improve their thermal and mechanical stability and can largely suppress or even completely avoid thermally induced damaging effects [13]. Consequently, oxide particle strengthened (ODS) thin film materials as demonstrated for Pt-Al${}_{2}$O${}_{3}$, Pt-Rh/NiO${}_{x}$, Pt-Rh/CoO${}_{x}$ and Pt-Rh/HfO${}_{x}$ [14,15] are already used in the SAW sensor technology above 350 ${}^{\circ }C$ because there have been no alternatives yet. A disadvantage of using noble metals is that they increase the device costs drastically. Hence in the past few years intensive research focusing on the search for alternative IDT thin films, which can offer long lifetimes and high reliability while lowering the device cost, has gained more impetus [16,17,18,19,20].

Rane et al. [16,17] and Seifert et al. [18,21,22] studied different W-Mo multilayer systems and RuAl thin films for SAW sensor applications along with the development of their morphology and electrical resistance on oxidized (100) Si and on high-temperature piezoelectric CTGS (Ca${}_{3}$TaGa${}_{3}$Si${}_{2}$O${}_{14}$) substrates. It was shown that oxidation, especially of tungsten which is accompanied by a significant change in relevant acoustic properties like density and stiffness, is a considerable disadvantage of the W-Mo multilayers if they are not covered by a suitable protection layer and are not separated from the substrate by a diffusion barrier layer. Pure molybdenum (T${}_{\mathrm{melt}}$ of bulk Mo: 2623 ${}^{\circ }C$. This and following data taken from Reference [23]) could also be a candidate for high-temperature IDTs due to its low electrical resistivity for a metal with high melting temperature. In comparison to platinum (T${}_{\mathrm{melt}}$ of bulk Pt: 1768 ${}^{\circ }C$) the agglomeration tendency is much lower since the activation energy for self-diffusion (bulk Mo ≈ 4.8 eV [24]) is much higher than for bulk Pt (≈ 3 eV [25]). In addition, the higher thermal conductivity of Mo (bulk Mo: 138 W m^−1^ K^−1^, bulk Pt: 71.6 W m^−1^ K^−1^, both at 300 K) lowers the risk of thermally induced local damages as thermo-migration in the electrode material or cracking or the formation of electric shorts due to local temperature gradients. Besides this, the electrical resistivity (bulk Mo $\rho^{Mo}\approx 5.5\;\mu \Omega$cm at 300 K) and the thermal expansion coefficient (bulk Mo $\alpha_{th}^{Mo}\approx 4.8\times 10^{-6}$ K${}^{-1}$ at 300 K) are much lower in Mo compared with Pt ($\rho^{Pt}\approx 10.8\;\mu \Omega$cm, $\alpha_{th}^{Pt}\approx 8.8\times 10^{-6}$ K${}^{-1}$ both at 300 K). Since $\alpha_{th}^{Mo}$ is closer to the value of CTGS ($\alpha_{th}^{CTGS}=4-5\times 10^{-6}$ K${}^{-1}$ in the basal plane at 300 K [26,27]) lower thermally induced mechanical stresses are expected in Mo-CTGS systems as compared to Pt on CTGS. All these properties make Mo based material systems very promising for the high-temperature SAW technology. However, pure Mo components in microdevices show a relatively low thermal stability and can fail even below 800 ${}^{\circ }$C as reported by Samanta et al. [28].

To overcome these disadvantages, various approaches can be applied for improving the high-temperature properties of a material for example, forming solid-solutions, strain or precipitation hardening, dispersion strengthening or grain size refinement. For instance for dispersion strengthening of bulk Mo materials oxide or carbide particles can be used as local barriers for diffusion and the movement of dislocations improving their thermo-mechanical properties [29]. An additional strengthening effect can result from blocking the grain growth or the formation of sub-microstructures during the thermal treatment based on the Hall-Petch relation ([30] and references therein). Thermally stable oxide particles are preferred to form oxide dispersion strengthened Mo material systems (ODS-Mo) using rare earth oxides. These ODS-Mo materials using La${}_{2}$O${}_{3}$ have received considerable attention especially due to their superior ductility, toughness and creep resistance properties at high temperatures and are currently used for high-temperature components [31,32,33,34]. For instance, Endo et al. found that the recrystallization temperature increases from 1100–1200 ${}^{\circ }$C for pure Mo wires to 1500–1600 ${}^{\circ }$C if La${}_{2}$O${}_{3}$ is added to Mo [33]. Bianco et al. studied the effect of La${}_{2}$O${}_{3}$, Y${}_{2}$O${}_{3}$ and CeO${}_{2}$ addition to a Mo matrix and observed that adding La${}_{2}$O${}_{3}$ resulted in a highest ultimate tensile strength and led to an increase of the creep rupture strength [34]. It was observed that the hardening effect obtained due to the addition of oxide particles was 3 to 5 orders of magnitude, so much more effective than what could be related to the reduced grain size (Hall-Petch relation). They also found that the amount of La${}_{2}$O${}_{3}$ in the Mo matrix significantly influences the material properties. The highest thermal stability was achieved if 0.6–1.5 wt.% La${}_{2}$O${}_{3}$ was added to Mo. However, intensive studies on ODS-Mo thin films with a La${}_{2}$O${}_{3}$ particle dispersion phase (ODS La${}_{2}$O${}_{3}$-Mo thin films) together with a suitable fabrication technology are still missing. Therefore, this paper studies the fabrication of La${}_{2}$O${}_{3}$-ODS molybdenum thin films by sputter deposition of thin Mo layers and extremely thin lanthanum interlayers forming molybdenum–lanthanum oxide multilayer systems on SiO${}_{2}$/Si substrates.

## 2. Materials and Methods

To produce the desired multilayer structures co-sputtering from both a lanthanum oxide and a molybdenum target to fabricate La${}_{2}$O${}_{3}$ strengthened Mo films is not really applicable, because La${}_{2}$O${}_{3}$ targets degrade very fast. Hence, a new approach for fabricating ODS La${}_{2}$O${}_{3}$-Mo thin films was evaluated which includes sequential multilayer co-sputtering of molybdenum and lanthanum. The multilayers were sputtered in a two target chamber from a pure Mo (99.95% Mo) and a pure La (99.95% La target). The deposition process comprises an in-situ oxidation process of the ultrathin La layers of a nominal thickness between 1 and 0.125 nm before the deposition of the subsequent Mo layer. It is assumed that the La layer growth can largely be described by the Volmer-Weber mechanism [35] forming a discontinuous layer of La islands in the initial steps of the layer deposition at a substrate temperature close to room temperature. As lanthanum can easily form either lanthanum oxide or lanthanum hydroxide it was found that the residual oxygen in the deposition chamber is sufficient to oxidize the lanthanum islands forming only La${}_{2}$O${}_{3}$ if water vapor is kept away in vacuum condition. In order to completely oxidize the deposited La islands the deposition process was interrupted after deposition of each La layer for a certain time (25 to 60 min depending on the La layer thickness) so as to ensure complete oxidation. This waiting time was determined based on prior tests as well as by monitoring the base pressure in the chamber. After La deposition, the waiting time was determined based on the time required for the pressure in the chamber to stabilize to the base pressure that the chamber had before deposition. This indirect method ensured that no more residual oxygen in the chamber was being taken up by either the La layer or the La target itself, thus indicating the complete formation of a lanthanum oxide film (see Section 3.1 for more details and X-ray photoelectron spectroscopy (XPS) studies.

**Figure 1:**
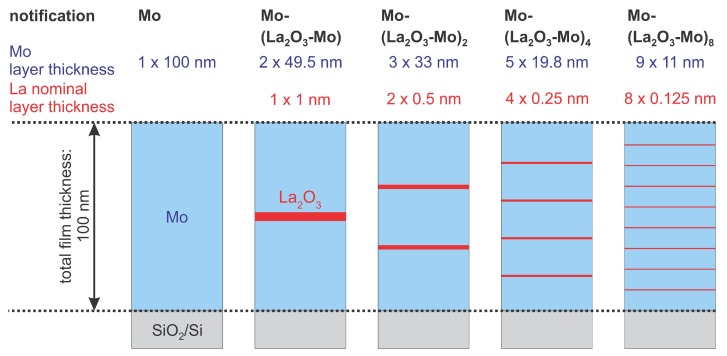
Sketch of the various types of samples with a different number *n* of La${}_{2}$O${}_{3}$ interlayers. For the multilayers the La nominal layer thickness is the thickness theoretically reached after 1/n of the deposition time necessary to deposit 1 nm of La. The Mo layer thickness is reached after 1/(n+1) of the time necessary to deposit 99 nm of Mo.

In order to achieve an electrical conductivity of the multilayers as high as possible, only a low content of insulating lanthanum oxide in the Mo matrix is allowed which however needs to be high enough to realize an effective strengthening of the Mo films as desired. Furthermore, it is well known from the literature that the thickness of the individual layers in pure Mo multilayer stacks (without lanthanum oxide) has a significant influence on the mechanical and electrical properties of the Mo multilayers due to the size effect [36]. Accordingly, for this study various film systems with up to 8 (Mo-La${}_{2}$O${}_{3}$) bilayers were prepared. In the following, the nomenclature Mo-(La${}_{2}$O${}_{3}$-Mo)${}_{n}$ is used to denote the layer configuration of the samples, where the first named Mo denotes the covering Mo layer and *n* represents the number of (La${}_{2}$O${}_{3}$-Mo) bilayers. The measured data of each multilayer system were compared with results observed for pure 100 nm Mo films. Figure 1 shows the architecture of all multilayer systems investigated in the present paper. The total thickness of the multilayer films was kept constant at 100 nm so that the thickness of the individual layers of Mo and La varied with the number of bilayers. The total thickness of La and Mo was 1 nm and 99 nm, respectively, that corresponds to a constant value of about 1 wt.% La${}_{2}$O${}_{3}$ in the Mo matrix which is in the range given in Reference [34].

The multilayers were deposited on cleaned (100) Si substrates with 1 μm thick thermally grown SiO${}_{2}$ on top with a sample size of (10×10) mm${}^{2}$. The sputtering was carried out in a high vacuum (HV) chamber of a dedicated cluster tool using matched DC generators (Magnetron Power Supply MP-2, Hüttinger Elektronik, Freiburg, Germany) and circular magnetrons (diameter 100 mm, Kurt J. Lesker Company, Jefferson Hills, PA, USA). Each magnetron source has its own pneumatically driven shutter system which is controlled by the computer of the cluster tool. A load lock chamber of the cluster permits to keep the targets in high vacuum conditions all the time to prevent oxidation of the target materials.

Before deposition, the substrates were kept in high vacuum at the base chamber pressure of ≈3 $\times 10^{-6}$ mbar for a constant time duration for each experiment. During the deposition, the chamber pressure was $1.6\times 10^{-3}$ mbar using Ar (purity 99.999% Ar) process gas with the flow rate of ≈30 sccm. The DC power was 500 W and 15 W for Mo and La, respectively. The substrate temperature was nearly room temperature. Before starting the experiments the deposition rate was determined under these conditions for both target materials being 29 nm min${}^{-1}$ and 2 nm min${}^{-1}$ for Mo and La, respectively, in the stationary state of the magnetron operation with a linear behavior of the deposition rate versus time. Therefore, before each deposition the conditions were stabilized for several minutes by depositing onto a closed shutter. The samples rotated at a constant speed of ≈10 rpm. The most relevant sputter parameters are summarized in Table 1.

After deposition of the multilayer films, the samples were annealed at 800 ${}^{\circ }$C in high vacuum conditions at a chamber pressure in the range of 10${}^{-5}$ mbar (to exclude oxidation effects of Mo) for 24 h, 48 h and 120 h. The films were characterized with respect to their microstructure and electrical behavior in the as-deposited state and after heat treatment using scanning (SEM, Zeiss Ultra Plus, Oberkochen, Germany) and transmission electron microscopy (TEM, Technai F30, FEI company, Hillsboro, OR, USA) with energy dispersive X-ray spectroscopy (EDX, Octane T Optima, EDAX Company, Mahwah, NJ, USA), X-ray diffraction (XRD, Philips X’Pert PW3040/00, CoKα), atomic force microscopy (AFM, Dimension Icon, Bruker, Billerica, MA, USA, and electrical sheet resistance measurements (van der Pauw technique, vdP). The X-ray photoelectron spectroscopy measurements (XPS) were done with a PHI 5600 CI system (Physical Electronics, Chanhassen, MN, USA) using non-monochromatic MgKα radiation.

## 3. Results

### 3.1. Composition of the La Interlayers

**Figure 2:**
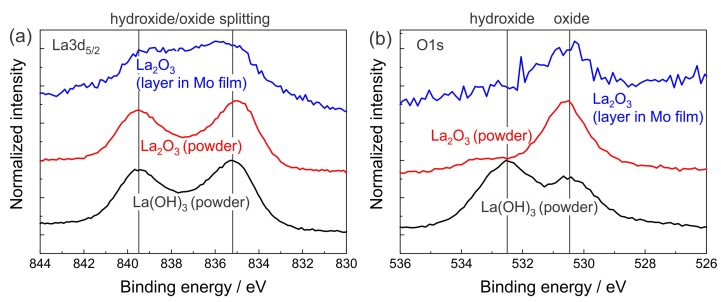
Results of the X-ray photoelectron spectroscopy (XPS) measurements of the (**a**) La3d${}_{5/2}$ and (**b**) O1s peak for a La(OH)${}_{3}$ powder reference, a La${}_{2}$O${}_{3}$ powder reference and the 2 nm La-X layer deposited between two 10 nm thick Mo layers. The results indicate that the La-X layer is composed of La${}_{2}$O${}_{3}$.

In a first step, the magnetron sputtered and subsequently oxidized La layers were investigated to ascertain their chemical composition. As mentioned above, lanthanum can form either lanthanum oxide (La${}_{2}$O${}_{3}$) or, if water vapor is present, lanthanum hydroxide (La(OH)${}_{3}$). Under vacuum conditions the formation of either La${}_{2}$O${}_{3}$ or La(OH)${}_{3}$ depends on the leakage rate and total pressure level of the process chamber including the residual water at the chamber walls as well as the purity of the Ar process gas (purity 99,999% Ar was used). Before each deposition process, both targets were pre-sputtered for a certain time with a closed shutter to remove contaminations from the surfaces of the targets and to reach a steady-state condition for the deposition. However, the chemical composition of the resulting La-based (La-X) films had to be verified under these conditions by XPS analysis. To provide reference La(OH)${}_{3}$ samples for the XPS analysis a La${}_{2}$O${}_{3}$ powder sample was stored for several hours in wet air to be sure that it converts to hydroxide. A second La${}_{2}$O${}_{3}$ powder sample was annealed at 800 ${}^{\circ }$C for 24 h in high vacuum to create a La${}_{2}$O${}_{3}$ standard. In addition, a three-layer system consisting of 10 nm Mo on 2 nm La on 10 nm Mo was deposited on a SiO${}_{2}$/Si substrate. The deposition process was interrupted for 25 min after the deposition of the 2 nm La layer. All samples were subsequently investigated with XPS by sputter depth-profiling with Ar. Figure 2 shows the results of the XPS measurements of the La3d${}_{5/2}$ and O1s peaks during depth profiling of the La-X interlayer region in the Mo-(La-X)-Mo layer in comparison with results measured on the powder references after 10 min of sputter cleaning.

The peak shape of the La3d${}_{5/2}$ peak is similar for both the La(OH)${}_{3}$ and the La${}_{2}$O${}_{3}$ powder (Figure 2a). The peak measured for the La-X layer is quite similar and its broadening can be explained by the very small thickness of this layer. The peak position of metallic La is at 836.8 eV and none of the measured samples shows this peak which proves that there is no metallic La in the La-X film. However, from the measurement of the La3d${}_{5/2}$ peak, it is not possible to distinguish between the lanthanum oxide and lanthanum hydroxide. This becomes possible by the analysis of the O1s peak. Lanthanum hydroxide is characterized by a high-energy O1s peak at 532.5 eV that is clearly visible in Figure 2b in case of the lanthanum oxide powder material which was stored for a long time in wet air and, thus converted to lanthanum hydroxide. This peak lacks in the spectrum of the lanthanum oxide, in which a strong peak appears at a lower energy (530.5 eV). The measurement of the La-X layer only shows an intensity at this lower energy, which indicates the formation of La${}_{2}$O${}_{3}$ in this interlayer.

The measured intensities also allow a quantitative estimation of the ratio between La and O in the samples. The measured ratio of La to O in the La interlayer region was about 30…40 to 70…60 that is close to that ratio found for the La${}_{2}$O${}_{3}$ reference powder material (ratio 30 at% La to 70 at% O, for comparison for La(OH)${}_{3}$: 20 at% La to 80 at% O).

Both results, the composition analysis as well as the absence of the hydroxide peak in Figure 2b prove that mainly lanthanum oxide is formed when a very thin La layer (thickness ≤2 nm) is deposited on top of a Mo layer with an interruption time of 25 min before the next Mo layer is added. Interpolating from this result, since a 2 nm La film was completely oxidized, it can be expected that films of a lower thickness (≤1 nm in the multilayers studied) should be completely oxidized as well.

### 3.2. Mo-(La${}_{2}$O${}_{3}$-Mo)${}_{n}$ Multilayers

#### 3.2.1. Microstructure

**Figure 3:**
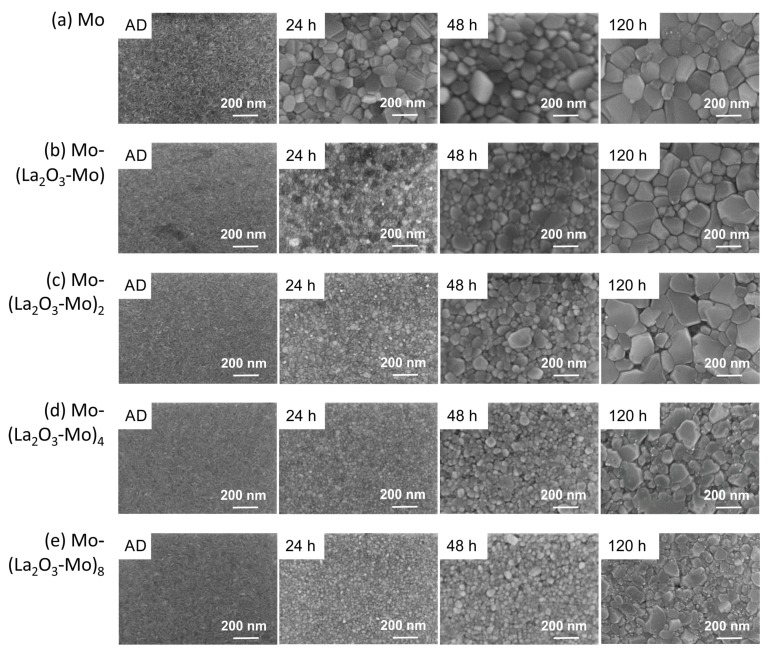
Scanning electron microscopy (SEM) micrographs (inLens, 20 kV) of Mo-(La${}_{2}$O${}_{3}$-Mo)${}_{n}$ multilayer systems on SiO${}_{2}$/Si in the as-deposited state (AD) and after thermal treatment for different time durations in high vacuum at 800 ${}^{\circ }$C (**a**) pure Mo, (**b**) Mo-(La${}_{2}$O${}_{3}$-Mo), (**c**) Mo-(La${}_{2}$O${}_{3}$-Mo)${}_{2}$, (**d**) Mo-(La${}_{2}$O${}_{3}$-Mo)${}_{4}$ and (**e**) Mo-(La${}_{2}$O${}_{3}$-Mo)${}_{8}$.

Figure 3 presents SEM images of the surfaces of the different Mo-(La${}_{2}$O${}_{3}$-Mo)${}_{n}$ multilayer systems. It can be seen that the multilayer stacks have a fine-grained polycrystalline microstructure after deposition with a grain size up to several ten nanometers. No cracks and pores are visible. The pure 100 nm Mo film in Figure 3a shows a typical morphology known for thin Mo films on SiO${}_{2}$/Si substrates in the as-deposited state [16,17,37]. In the as-deposited state (AD, left column in Figure 3b–e), the morphology of the covering Mo layers in all the multilayers, independent of the number of layers, is similar to that of pure Mo (Figure 3a left).

Upon annealing, strongly differing surface morphologies show up in all the films with increasing annealing time. In case of the pure Mo film, initially the grain size increases significantly upon annealing for 24 h followed by a slower growth up to 120 h. In contrast, all the multilayer films exhibit much smaller grain sizes after annealing for 24 h than that observed for the pure Mo film. The grain size increase during annealing is significantly reduced with an increasing number of bilayers, indicating that the La${}_{2}$O${}_{3}$ distribution influences the grain growth. This is due to the pinning effect of the La${}_{2}$O${}_{3}$ interlayers (as explained below).

**Figure 4:**
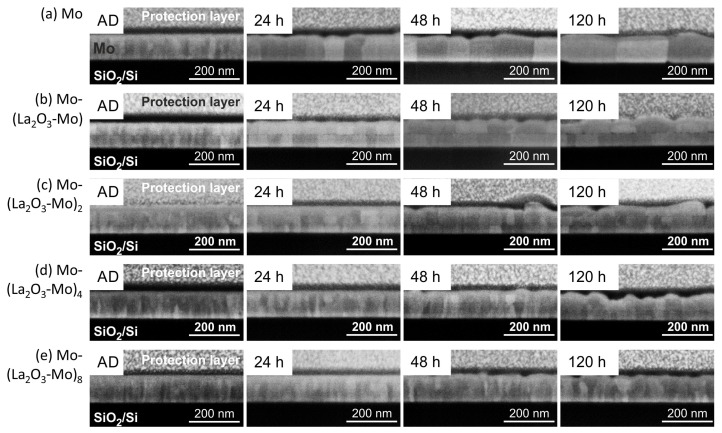
SEM micrographs (inLens, 20 keV) of FIB cross-sections of the Mo-(La${}_{2}$O${}_{3}$-Mo)${}_{n}$ multilayer systems on SiO${}_{2}$/Si in the as-deposited state (AD) and after thermal treatment in high vacuum for different time durations at 800 ${}^{\circ }$C (**a**) pure Mo, (**b**) Mo-(La${}_{2}$O${}_{3}$-Mo), (**c**) Mo-(La${}_{2}$O${}_{3}$-Mo)${}_{2}$, (**d**) Mo-(La${}_{2}$O${}_{3}$-Mo)${}_{4}$ and (**e**) Mo-(La${}_{2}$O${}_{3}$-Mo)${}_{8}$. For FIB cross-sectioning the samples were covered at first with electron beam induced carbon (black layer on top of the multilayer film) and then with ion beam induced Pt inside the FIB workstation.

Figure 4 summarizes the SEM micrographs of the FIB (Focussed Ion Beam) cross sections of the multilayer systems. The interface between the Mo and La${}_{2}$O${}_{3}$ layer can be clearly identified only in the Mo-(La${}_{2}$O${}_{3}$-Mo) multilayer system (Figure 4b) in the as-deposited state as well as after all the annealing durations. This interface is not detectable in the other multilayer systems for thinner La${}_{2}$O${}_{3}$ layers. Upon annealing for 24 h, the out-of-plane structure is slightly clearer for all the films. The pure Mo film shows drastically grown columnar grains that span the entire film thickness. In the Mo-(La${}_{2}$O${}_{3}$-Mo) film, columnar grains extending only up to midway of the film thickness can be seen indicating an interruption of the grain growth due to the La${}_{2}$O${}_{3}$ interlayer. In contrast, the multilayers are found to be composed of grains that are small, tapered and of different heights along with some grains that also span the entire thickness. With increasing annealing time, as seen in the top-surface view, the multilayer film with the highest number of La${}_{2}$O${}_{3}$ layers exhibits the lowest in-plane grain growth. It can also be observed that with an increasing number of La${}_{2}$O${}_{3}$ layers, the in-plane grain size dispersion becomes narrower. The Mo-La${}_{2}$O${}_{3}$-Mo layer is composed of some extremely large and some very small grains while a more uniform grain size is seen in Mo-(La${}_{2}$O${}_{3}$-Mo)${}_{8}$.

**Figure 5:**
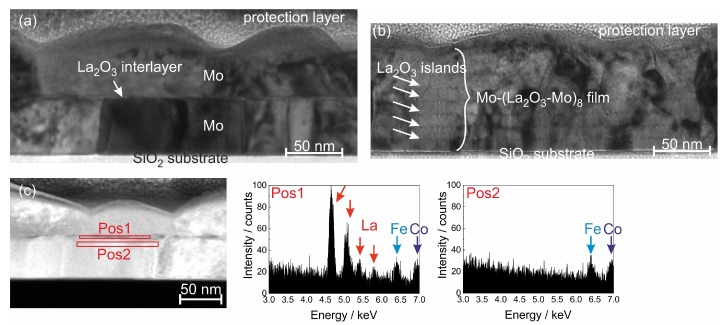
Transmission electron microscopy (TEM) study on Mo-(La${}_{2}$O${}_{3}$-Mo)${}_{n}$ multilayer systems deposited on SiO${}_{2}$/Si after annealing at 800 ${}^{\circ }$C for 120 h in high vacuum: bright field TEM image of FIB cross-section lamellas of (**a**) a Mo-(La${}_{2}$O${}_{3}$-Mo) and (**b**) a Mo-(La${}_{2}$O${}_{3}$-Mo)${}_{8}$ film, (**c**) Scanning transmission electron microscopy image (predominant chemical contrast) together with energy dispersive X-ray spectroscopy (EDX) analyses of Mo-(La${}_{2}$O${}_{3}$-Mo). The Fe and Co signals are caused by the TEM device.

The layer structure and especially the (La${}_{2}$O${}_{3}$-Mo) interface between two adjacent Mo layers were studied more in detail using transmission electron microscopy (TEM). In Figure 5a,b as two examples bright field TEM images of the Mo-(La${}_{2}$O${}_{3}$-Mo) and Mo-(La${}_{2}$O${}_{3}$-Mo)${}_{8}$ multilayer after annealing at 800 ${}^{\circ }$C for 120 h in HV are presented.

In the Mo-(La${}_{2}$O${}_{3}$-Mo) film, a clear interruption of the grain growth is observed midway of the film thickness where the presence of La${}_{2}$O${}_{3}$ was confirmed by EDX (Figure 5c also shows the good localization of La between the individual Mo layers). The discontinuity in grain growth is observed throughout the length of the film which is attributed to the presence of a completely closed continuous layer of La${}_{2}$O${}_{3}$ that interrupts the grain growth of Mo in the out-of-plane direction (see also Figure 4b 120 h).

In case of the Mo-(La${}_{2}$O${}_{3}$-Mo)${}_{8}$ film, the layer structure is still visible (Figure 5b). However, it can be seen that the La${}_{2}$O${}_{3}$ layers are discontinuous and formed of an arrangement of distinctly separated particles. Unlike the Mo-(La${}_{2}$O${}_{3}$-Mo) film, herein along with discontinuous grains, several columnar grains of Mo extending across several sublayers or completely through the film thickness can also be seen (Figure 4e 120 h). This could be due to the discontinuous La${}_{2}$O${}_{3}$ layers. Due to the low layer thickness of La${}_{2}$O${}_{3}$ it can be expected that the layers consist of La${}_{2}$O${}_{3}$ islands which are well separated from each other as is typically observed for the nucleation of islands in the early stages of film growth [35]. Thus, it can be expected that the out-of-plane grain growth is likely to be interrupted only at locations around La${}_{2}$O${}_{3}$ particles while at other locations the grains can grow continuously.

The in-plane grain size also reduces with an increasing number of bilayer periods which could be attributed to the presence of La${}_{2}$O${}_{3}$ at the top and bottom surfaces of the Mo grains and probably also at the adjacent grain boundaries (triple junctions), pinning the grains in multiple directions. Another reason for decreased in-plane grain size could be a result of a mixture of two types of grains that are interspersed, that is, uninterrupted grains (columnar, spanning multiple Mo layers) and grains that are pinned by La${}_{2}$O${}_{3}$ (interrupted across layers). The probability of this mixture of grains being more uniformly dispersed increases with increasing number of bilayer periods. With a higher number of La${}_{2}$O${}_{3}$ layers, a better uniformity of La${}_{2}$O${}_{3}$ particles throughout the film was achieved. This could also explain the more uniform in-plane grain size in the Mo-(La${}_{2}$O${}_{3}$-Mo)${}_{8}$ film.

The EDX measurements also reveal that there is no oxidation of the Mo at the sample surface even after annealing for 120 h at 800 ${}^{\circ }$C in HV. Compared to other systems which are investigated concerning their suitability for high temperature application in SAW devices the oxidation resistance is strongly improved. In case of RuAl thin films on thermally oxidized Si substrates a 20 nm thick Al${}_{2}$O${}_{3}$ layer is formed on top of the sample during annealing at 800 ${}^{\circ }$C for only 10 h in high vacuum [18], while in Ti-Al based films a strong oxidation of the Al takes place even if an AlN cover layer is applied [38].

#### 3.2.2. Phase Formation

**Figure 6:**
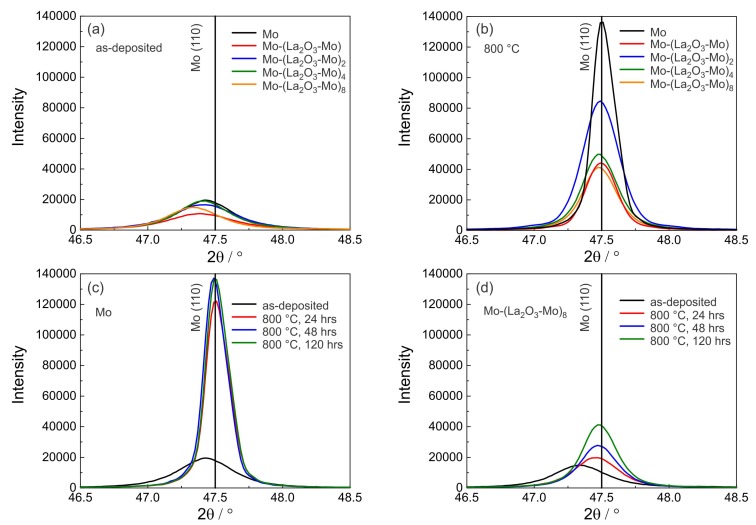
X-ray diffraction (XRD) pattern (range around the Mo (110) peak position) of the pure Mo and the Mo-(La${}_{2}$O${}_{3}$-Mo)${}_{n}$ films (Bragg-Brentano geometry, Co-Kα), (**a**) in the as-deposited state and (**b**) after thermal treatment in high vacuum at 800 ${}^{\circ }$C for 120 h. (**c**) Pure Mo and (**d**) the Mo-(La${}_{2}$O${}_{3}$-Mo)${}_{8}$ multilayer system, both in as-deposited state and annealed at 800 ${}^{\circ }$C for 24 h, 48 h and 120 h.

The XRD measurements show that the Mo and the Mo-(La${}_{2}$O${}_{3}$-Mo)${}_{n}$ layers in the as-deposited state are polycrystalline and exhibit XRD peaks associated with the body centered cubic (bcc) crystal structure of molybdenum (powder reference). The films grow with a dominant (110) orientation. Figure 6a,b show the diffractograms around the Mo (110) peak position for the different samples in the as-deposited state and after annealing at 800 ${}^{\circ }$C for 120 h. The position of the (110) Mo peak at 47.5${}^{\circ }$ (Co-K${}_{\alpha }$) of the powder Mo material is marked as a reference by a vertical line. In the as-deposited state, there is a low peak intensity and the Mo peak position is slightly shifted to lower values as compared to the reference (Figure 6a). After annealing the peak intensities are strongly increased as compared to the as-deposited state and the Mo peak position shifts to the theoretical value of the Mo powder material. The highest intensity is reached for the pure Mo film which is in agreement with the presence of the largest grains for this sample as visible from the cross-section images (Figure 4).

The results of the XRD measurements for the pure Mo film and the Mo-(La${}_{2}$O${}_{3}$-Mo)${}_{8}$ multilayer system for the different annealing times are presented in Figure 6c,d. For the pure Mo films in Figure 6c, the peak intensity compared to the as-deposited state strongly increases after annealing for 24 h but longer annealing times lead only to a small further increase of the peak intensity. The peak intensities in Figure 6c measured after annealing of pure Mo are significantly higher than those for the Mo-(La${}_{2}$O${}_{3}$-Mo)${}_{8}$ multilayer films in Figure 6d, which is explained by the larger grain size of pure Mo films compared with that of the Mo-(La${}_{2}$O${}_{3}$-Mo)${}_{n}$ multilayers, as already seen in Figure 4. For the Mo-(La${}_{2}$O${}_{3}$-Mo)${}_{8}$ multilayer films (and likewise for all the other multilayer systems) a continuous increase in peak intensity with annealing time is observed (Figure 6d).

#### 3.2.3. Roughness

**Figure 7:**
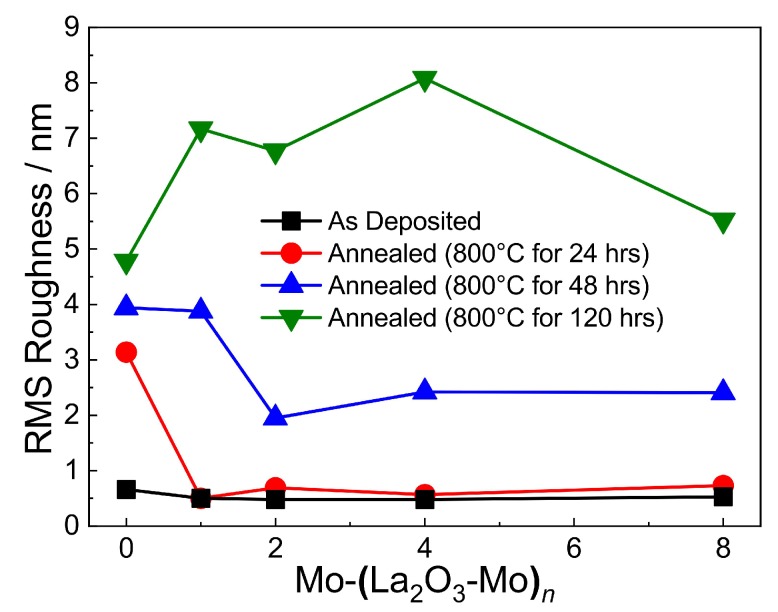
Root mean square (RMS) roughness of the sputtered Mo-(La${}_{2}$O${}_{3}$-Mo)${}_{n}$ multilayers on SiO${}_{2}$/Si as a function of the number *n* of (La${}_{2}$O${}_{3}$-Mo) bilayers for different annealing time durations.

Diffuse scattering of electrons at surfaces is more pronounced the higher the roughness is, that is, the electrical sheet resistance generally increases with roughness, especially for thin films. The root mean square roughness (RMS) of the multilayer systems shown in Figure 7 was obtained on a constant measurement area of 2 μm × 2 μm. The RMS was found to be less than 1 nm for all the as-deposited films. In contrast to the multilayer films, the roughness of the pure Mo films already strongly increases after annealing for 24 h. The RMS values of the multilayers increase after annealing at 48 and 120 h. For all multilayers annealed for 120 h, the Mo-(La${}_{2}$O${}_{3}$-Mo)${}_{8}$ sample has the lowest roughness, because the thickness of the individual Mo layers decreases and thermally induced grain growth is blocked by the La${}_{2}$O${}_{3}$ islands. The smaller grains result in a lower roughness. The AFM results are consistent with the morphology observed in the SEM micrographs in Figure 3 and Figure 4.

#### 3.2.4. Electrical Resistivity

**Figure 8:**
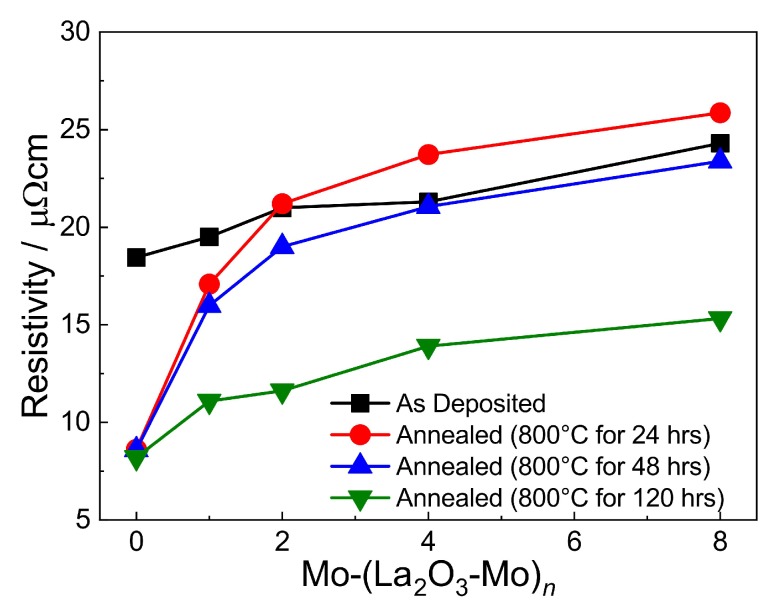
Determined resistivity of the 100 nm thick Mo and Mo-(La${}_{2}$O${}_{3}$-Mo)${}_{n}$ thin film systems on SiO${}_{2}$/Si based on the measured sheet resistances using the van der Pauw method.

Figure 8 summarizes the measured resistivity values of the Mo-(La${}_{2}$O${}_{3}$-Mo)${}_{n}$ multilayer systems. In the as-deposited state the resistivity of the 100 nm thick pure Mo layer deposited on SiO${}_{2}$/Si is approximately 18.5 μΩcm. For the multilayer systems an integral resistivity for the whole system containing also insulating La${}_{2}$O${}_{3}$ as a layer or individual particles is determined. The layers act as a parallel circuit of the continuous (conductive) Mo layers shunted or interrupted by more or less discontinuous interlayers of La${}_{2}$O${}_{3}$ particles. Anyhow, this measured overall resistivity of the film system can be used as a basis to design electronic devices for example, based on SAW structures.

Generally, resistivity values in the as-deposited state of the multilayer systems are found to increase with the number *n* of (La${}_{2}$O${}_{3}$-Mo) bilayers that is, with decreasing the individual Mo layer thickness. This tendency is coherent with theoretical models of thin film resistivity since the resistivity is not only affected by an increasing grain boundary volume and a higher number of defects and impurities when the number of interfaces increases but also by electron scattering at the La${}_{2}$O${}_{3}$-Mo layer interfaces [39,40,41]. The effect of the mean free path of electrons (MFPE) of 39.5 nm of Mo [42] on the electrical resistivity should become predominant as the thickness of the Mo layer reaches this limit (i.e., for all films with n≥2). Then the size effect becomes dominant because the thickness of the individual Mo layers and the in-plane grain size reaches the MFPE value. Upon annealing for 24 h, the resistivity of the pure Mo layers reduces drastically but there is only a slight decrease thereafter upon further annealing up to 120 h. In contrast, for the Mo-(La${}_{2}$O${}_{3}$-Mo)${}_{n}$ multilayers, the reduction of the resistivity is much lower for the annealing duration of 24 h (for Mo-(La${}_{2}$O${}_{3}$-Mo)) or it even increases slightly (for Mo-(La${}_{2}$O${}_{3}$-Mo)${}_{2-8}$). However, a more significant and comparable decrease is observed for the long annealing time of 120 h. The resistivity of the 100 nm Mo layer on SiO${}_{2}$/Si reaches a value of 8.2 μΩcm due to the observed distinct grain coarsening and defect annihilation. This value is close to that of pure Mo bulk material (≈5.57 μΩcm [43]).

It can be seen that the resistivity of the films increases with an increasing number of interlayers. A closer look at the increase reveals, that a higher number of layers which are extremely thin did not affect the resistivity increase with respect to the MFPE as it would be expected according to the theory of Fuchs-Sondheimer [40,41]. As the La${}_{2}$O${}_{3}$ interlayers do not form a continuous layer for n>2, the Fuchs-Sondheimer theory is not strictly applicable. Furthermore, the resistivity change can be attributed to the special microstructure of the multilayer films composed of a grain distribution of different heights and widths. As seen in Section 3.2.1 more uniformity in grain dimensions was obtained with an increasing number of La${}_{2}$O${}_{3}$ interlayers, thus increasing the number of interlayers helps to realize a more uniform microstructure. The resistivity in this case is governed by the grain size and the content of La${}_{2}$O${}_{3}$ in the film.

## 4. Conclusions

The present paper presents a study of different Mo-(La${}_{2}$O${}_{3}$-Mo)${}_{n}$ multilayer thin film systems which were deposited by magnetron sputtering on thermally oxidized (100) Si substrates to form ODS La${}_{2}$O${}_{3}$-Mo thin films for future applications in high-temperature wireless SAW devices. The multilayer thin films were investigated in the as-deposited state and after thermal treatment at 800 ${}^{\circ }$C up to 120 h in high vacuum conditions. A content of about 1 wt.% La${}_{2}$O${}_{3}$ was distributed through the Mo-(La${}_{2}$O${}_{3}$-Mo)${}_{n}$ multilayer systems, which were covered with a pure Mo film. The total thickness of the multilayer systems was kept constant at 100 nm for all the multilayer configurations varying the nominal thicknesses of the individual La and Mo layers from 0.125 to 1 nm and from 11.0 to 49.5 nm, respectively. La${}_{2}$O${}_{3}$ was formed by a waiting time of 25 to 60 min after the deposition of each La layer. To apply the investigated multilayer systems to SAW devices based on for example, high temperature stable CTGS or Langasite [44] substrates we expect that diffusion barrier layers are essential to prevent degradation effects. Also the influence of an electrical power load on structured electrode lines needs to be investigated for long term applications. Furthermore, the following conclusions are derived:

- All Mo-(La${}_{2}$O${}_{3}$-Mo)${}_{n}$ multilayers deposited at room temperature (RT) are polycrystalline and free of significant cracks and they exhibit a similar morphology in the as-deposited state.
- In all multilayers the in-plane grain size reduces with an increasing number of bilayers and, thus decreasing thickness of the Mo layers, both in as-deposited state and after thermal treatment.
- There is no clear trend in the RMS roughness with the number *n* of (La${}_{2}$O${}_{3}$-Mo) bilayers. However, the roughness upon annealing at 800 ${}^{\circ }$C for 120 h is lowest for the Mo-(La${}_{2}$O${}_{3}$-Mo)${}_{8}$ system.
- The influence of the increase in RMS roughness during the annealing on the electrical resistivity is overcompensated by defect annihilation.
- Annealing of the multilayer systems at 800 ${}^{\circ }$C for 120 h leads to a reduction in the resistivity due to grain coarsening which results in reduced electron scattering. The annealed Mo layers show the lowest resistivity values of ≈8.2 μΩcm. For the Mo-(La${}_{2}$O${}_{3}$-Mo)${}_{n}$ multilayer films with n=4 and n=8 (La${}_{2}$O${}_{3}$-Mo) bilayers a slight increase in resistivity is observed after annealing at 800 ${}^{\circ }$C for 24 h compared to the as-deposited state.
- The results show that the multilayer systems retain a clearly visible periodic structure of Mo and La${}_{2}$O${}_{3}$ along the growth direction even after annealing at 800 ${}^{\circ }$C for 120 h.
- In case of 1 nm thickness of the La deposition, a continuous and closed La${}_{2}$O${}_{3}$ layer was formed on top of Mo. Thus the La${}_{2}$O${}_{3}$ in Mo-(La${}_{2}$O${}_{3}$-Mo) provided a layer of complete chemical and physical discontinuity to the Mo layers. However, it was shown that extremely thin La interlayers (<1 nm) were present as a discontinuous layer of La${}_{2}$O${}_{3}$ particles.
- The results indicate that Mo-(La${}_{2}$O${}_{3}$-Mo)${}_{n}$ multilayer films can be appropriate material systems for IDT electrodes for applications in the high-temperature range above 600 ${}^{\circ }$C.

## Figures and Tables

**Table 1:** Sputter parameters used for the deposition of the Mo-(La${}_{2}$O${}_{3}$-Mo)${}_{n}$ multilayers.

Parameter	Unit	Quantity	
		**Mo**	**La**
DC power	W	500	15
Ar gas flow	sccm	30	
base pressure	mbar	$3\times 10^{-6}$	
pressure during deposition	mbar	$1-2\times 10^{-3}$	
substrate temperature	${}^{\circ }$C	≈25 (RT, not controlled)	
deposition time (nominal thickness)	s	104 (49.5 nm)	35 (1 nm)
		69 (33.0 nm)	17 (0.5 nm)
		42 (19.8 nm)	9 (0.25 nm)
		23 (11.0 nm)	4 (0.125 nm)
total film thickness	nm	100	
time delay after La was deposited	min	-	60 (1 nm)
			45 (0.5 nm)
			30 (0.25 nm)
			25 (0.125 nm)

## Abbreviations

The following abbreviations are used in this manuscript:

AD	as deposited
AFM	atomic force microscopy
bcc	body centered cubic
MFPE	mean free path of electrons
FIB	focussed ion beam
IDT	interdigital transducer
ODS	oxide particle strengthened
RMS	root mean square roughness
RT	room temperature
SAW	surface acoustic wave
SEM	scanning electron microscopy
TEM	transmission electron microscopy
vdP	van der Pauw
XPS	X-ray photoelectron spectroscopy
XRD	X-ray diffraction
